# Growth of Veterinary Literature

**Published:** 1895-06

**Authors:** 


GROWTH OF VETERINARY LITERATURE.—Perhaps in no one direction has the English-speaking veterinarian been more favored the past few years (especially the last two) than by the growth of veterinary literature. It might be equally well said, on the other hand, perhaps, that no nation was so ill-supplied with modern scientific veterinary literature as English-speaking countries. America, it seems, like every other nation, has had to pass through the kindergarten stage, or age of transformation, when all the literature had a double destiny, that of being so written and prepared to serve equally well the horseman or horse-owner and the veterinarian.

The growth and development of the schools found a serious difficulty confronting them in the sparsity of works to advise the student to secure for consultation and reading, and it seemed a long wait for the situation to be changed; but at last came the dawn of a better period, and we can now predict in the future the time when English-reading veterinarians will, like their foreign brethren, commence the process of selection of those works specially adapted for their particular line of work.

With this number we briefly review the many valuable additions to our store in the various branches of our work, and feel justly proud of this opportunity to accord the various authors our meed of praise for their labors, that have so much enriched us in the truest worth of any nation or people.

The Western Pennsylvania Veterinary Medical Association has ceased to hold meetings. As an important veterinary centre with many excellent practitioners this should not be, and we trust an earnest effort will be made to reorganize and take up the good work that awaits their doing and commands their responsibility.

Our readers' attention is called to the well-written letter to one of the editors of this Journal from that active, earnest, progressive veterinarian, Dr. Williams, of Montana, and for its publication there need be no apology.

